# Two New Rhabdoviruses (*Rhabdoviridae*) Isolated from Birds During Surveillance for Arboviral Encephalitis, Northeastern United States

**DOI:** 10.3201/eid0806.010384

**Published:** 2002-06

**Authors:** Amelia P.A. Travassos da Rosa, Thomas N. Mather, Tsutomu Takeda, Chris A. Whitehouse, Robert E. Shope, Vsevolod L. Popov, Hilda Guzman, Lark Coffey, Tais P. Araujo, Robert B. Tesh

**Affiliations:** * University of Texas Medical Branch, Galveston, Texas, USA; † University of Rhode Island, Kingston, Rhode Island, USA

**Keywords:** arbovirus surveillance, rhabdovirus, vesiculovirus, West Nile virus, virus identification, avian viruses, Rhode Island virus, Farmington virus

## Abstract

Two novel rhabdoviruses were isolated from birds during surveillance for arboviral encephalitis in the northeastern United States. The first, designated Farmington virus, is a tentative new member of the *Vesiculovirus* genus. The second, designated Rhode Island virus, is unclassified antigenically, but its ultrastructure and size are more similar to those of some of the plant rhabdoviruses. Both viruses infect birds and mice, as well as monkey kidney cells in culture, but their importance for human health is unknown.

 Since the appearance of *West Nile virus* (WNV) in North America in 1999 (*1*), interest in surveillance of bird mortality has heightened among epidemiologists and other public health personnel (*2*). This interest is based on recent experience indicating that surveillance of bird deaths, especially of crows and other members of the family *Corvidae*, is a sensitive method for detecting WNV activity in a region (*2*–*5*). Consequently, many public health diagnostic laboratories in the United States are now actively testing dead birds for WNV. We describe two new rhabdoviruses that were isolated from birds during surveillance studies for WNV and *Eastern equine encephalitis virus* (EEEV) activity in the northeastern United States. This finding serves as a reminder that WNV and EEEV are not the only viruses that may be associated with bird deaths in this region.

## Methods

### Viruses Studied

We examined three virus isolates from birds. Virus strains RI-166 and RI-175 were both isolated from brain tissue of dead pigeons (*Columba livia*) collected at two localities in Rhode Island in summer 2000, as part of WNV surveillance activities. The two dead pigeons were collected on September 15 and 16 in Barrington, Bristol County (#175), and East Providence, Providence County (#166), respectively. No trauma or obvious gross pathology was noticed in the brain of either bird at necropsy. Brain tissue, including nearly equal portions of cerebrum and cerebellum, was collected and immediately frozen at –80^o^C until processed for culture. Frozen tissue was thawed, and a small portion was completely homogenized in 3.0 mL of medium 199 (Sigma, St. Louis, MO). Homogenized brain tissue was centrifuged at 3,500 × *g* for 20 minutes at 2^o^C in a refrigerated centrifuge; then 100 μL of the supernatants was immediately added to 25-mL flasks containing Vero cell monolayers. Tissue cultures were incubated at 37^o^C and 5% CO_2_ and examined for cytopathic effect (CPE) on days 3–7 postinoculation.

The third virus, designated CT-114, was originally isolated from an unknown wild bird captured in central Connecticut in 1969 by the late Robert B. Wallis, during surveillance for EEEV (*6*). The original isolation of CT-114 virus was made by intracerebral injection of newborn mice; no other information is available about this isolate.

### Antigens and Immune Reagents

Antigens for the three virus unknowns were prepared from infected newborn mouse brain by the sucrose-acetone extraction method (*7*). Hyperimmune mouse ascitic fluids (HMAF) to RI-166 and CT-114 viruses were prepared in adult mice as described (*8*). The adult mouse immunization schedule was four intraperitoneal injections per week of 10% crude suspensions of infected suckling mouse brain in phosphate-buffered saline mixed with Freund’s adjuvant. To induce ascites formation, sarcoma 180 cells were given intraperitoneally with the final injection.

Of the other rhabdovirus antigens and immune reagents used to characterize the three virus unknowns, some antigens were sucrose-acetone–extracted infected mouse brain, while others were medium from infected Vero cell cultures. The latter viruses, antigens, and HMAF were from the Arbovirus Reference and Reagent Collection maintained at the University of Texas Medical Branch (UTMB).

### Serologic Tests

Complement fixation (CF) tests were performed by a microtechnique (*7*) with two full units of guinea pig complement. Titers were recorded as the highest dilutions giving 3+ or 4+ fixation of complement on a scale of 0 to 4+.

Indirect immunofluorescent antibody (IFA) tests were done on Vero and mosquito cells grown in eight-chamber Lab Tek tissue culture slides (Nunc, Inc., Naperville, IL). The mosquito cells tested were the C6/36 clone of *Aedes albopictus* cells (*9*) and a *Culex quinquefasciatus* cell line (*10*). After addition of virus, the Vero and mosquito cells were incubated with appropriate media at 37^o^C and 28^o^C, respectively. Culture slides with Vero cells were fixed in cold acetone when the cells showed 2+ to 3+ viral CPE; the mosquito cells were fixed after 6 days of incubation. The IFA tests were performed by using HMAF at dilutions of 1:10 and 1:20 and a commercial fluorescein isothiocyanate-conjugated goat antimouse immunoglobulin G (Sigma, St. Louis, MO) (*11*).

### Transmission Electron Microscopy

Immediately after removal of the medium, Vero cell monolayers infected with RI-175 and CT-114 viruses were fixed in a mixture of 1.25% formaldehyde and 2.5% glutaraldehyde in 0.05 M cacodylate buffer at pH 7.3, to which 0.03% trinitrophenol and 0.03% CaCl_2_ were added, as described (*12*). After primary fixation, monolayers were washed in cacodylate buffer. Then the cells were scraped off the plastic, pelleted by light centrifugation in buffer, and postfixed in 1% OsO_4_ in the same buffer. They were stained en bloc with 1% uranyl acetate in 0.1 M maleate buffer at pH 5.0, dehydrated in ethanol, and embedded in Poly/Bed 812 (Polysciences, Warrington, PA). Ultrathin sections were cut on a Reichert/Leica Ultracut S ultramicrotome (Leica Microsystems, Inc., Bannockburn, IL), stained with 2% aqueous uranyl acetate and 0.4% lead citrate, and examined with Philips 201 or Philips CM-100 electron microscopes at 60 kV (Philips Electron Optics, Eindhoven, the Netherlands).

## Results

### Biological Characteristics

Viruses RI-166 and RI-175 were initially isolated in cultures of Vero cells at the Center for Vector-Borne Disease (CVBD), University of Rhode Island. Media from the positive cultures were tested by immunoassay for WNV, EEEV, *Highlands J virus*, Jamestown Canyon virus, La Crosse virus, *Saint Louis encephalitis virus*, and Flanders virus antigens, with specific monoclonal and polyclonal antibodies; results were negative. Both viruses RI-166 and RI-175 were subsequently sent to UTMB for further study and characterization. When added to Vero cell cultures, both viruses produced extensive CPE within 48 hours. Newborn Institute for Cancer Research outbred mice that were injected intracerebrally with both RI-166 and RI-175 viruses became sick and moribund within 96 hours. RI-166 virus was also added to cultures of C6/36 and *Cx. quinquefasciatus* cells; it did not produce CPE, and no viral antigen could be detected in the mosquito cells when examined by IFA 6 days later.

Virus CT-114 was initially isolated by Robert Wallis at the Department of Epidemiology and Public Health, Yale University School of Medicine, following intracerebral injection of a homogenate of bird tissue into newborn mice. The virus was subsequently transferred to UTMB. Virus CT-114 produced illness and death in newborn mice 24–48 hours after intracerebral injection, as well as massive CPE in Vero cells within 48 hours; however, it did not produce CPE in the mosquito cells. Specific viral antigen was detected by IFA in *Cx. quinquefasciatus* cells injected with CT-114 virus, but not in C6/36 cells.

### Ultrastructure of Isolates

Virions of isolate CT-114 were bullet shaped and were found budding mostly into the intracytoplasmic vacuoles, either as single virions into a small vacuole, or as several virions budding into the same large vacuole (Figure, A and B). Virions of CT-114 were 55 nm–60 nm in diameter and 145 nm–150 nm long, with a periodicity of striations of 10.5 nm (Figure, B).

**Figure:**
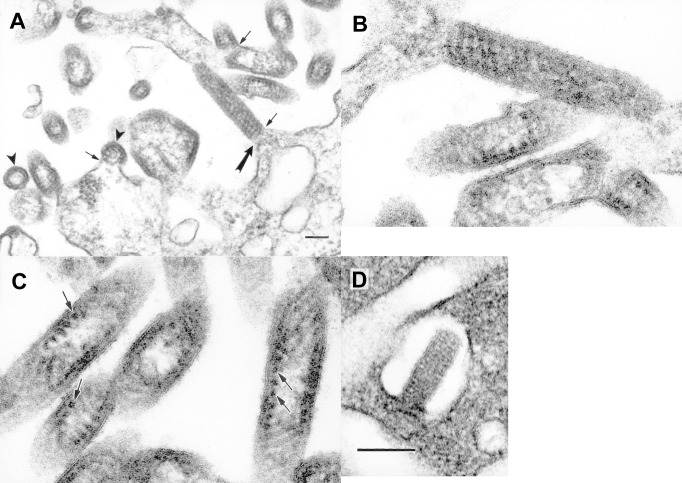
Ultrastructure of the new rhabdoviruses in infected Vero cells. A. Virions of isolate RI-175 budding from the surface of a Vero cell and from cell surface projections (arrows). Arrowheads mark cross-sections of virions. The virion indicated with a large arrow is enlarged in B. B. A virion of isolate RI-175 budding from host cell plasmalemma into an extracellular space. C. Details of the virion ultrastructure of isolate RI-175, showing spiral packaging of the nucleocapsid and its tubular structure in the cross-sections (arrows). D. A virion of the isolate CT-114 budding into an intracytoplasmic vacuole. Bar = 100 nm.

Virions of the isolate RI-175 were seen budding predominantly into the extracellular space from the plasmalemma of the Vero cells (Figure, C and D). The virions were bacilliform, measuring 90 nm–100 nm in diameter, up to 500 nm long, and with a 20- to 25-nm periodicity of striations. In some cross-sections, the spiral packaging of the nucleocapsid could be seen and had the appearance of tubules 9 nm in diameter (Figure, C). Large groups of virions could be observed outside the cells.

### Antigenic Characteristics

On the basis of their rhabdovirus-like morphology, RI-166, RI-175, and CT-114 antigens and HMAFs were examined by CF against 36 rhabdovirus antigens and HMAFs in our reference collection. The 36 agents included *Carajas virus;* *Chandipura virus*; *Cocal virus;* *Isfahan virus*; *Maraba virus*; *Piry virus;* vesicular stomatitis virus, types Alagoas, Indiana, and New Jersey; *Vesiculovirus* species Boteke, Jurona, Klamath, La Joya, Malpais Spring, Radi, and Yug Bogdanovac; *Iriri virus,* Flanders virus, Mosqueiro virus, Mossuril virus, Kern Canyon virus, Nkolbisson virus, Le Dantec virus, Connecticut virus, New Minto virus, sawgrass virus, Chaco virus, Timbo virus, Bangoran virus, Inhangapi virus, Joinjakaka virus, Kannamangalam virus, Kotonkan virus, Marco virus, Tibrogargan virus, and Yata virus (*13*,*14*).

In addition, RI-166 antigen was also tested against 26 other rhabdovirus HMAFs: Calchaqui, Gray Lodge, Kwatta, Mount Elgon bat, Perinet, Porton, Duvenhage, Lagos bat, Mokola, Rabies, Bahia Grande, Hart Park, Kamese, Keuraliba, Almpiwar, Aruac, Bimbo, Charleville, Coastal Plains, Gossas, Kolongo, Navarro, Obodhiang, Parry Creek, Rio Grande, and Sandjimba. In CF tests, RI-166 (selected as the prototype) and RI-175 viruses were indistinguishable (Table 1); but RI-166 antigen and HMAF did not react with any of the other rhabdovirus antigens or HMAFs listed. Because of the geographic region where they were isolated, we initially suspected that RI-166 and CT-114 might be Connecticut or Flanders viruses. However, no antigenic relationship was shown by CF test (Table 1). The antigen RI 907-36 was prepared from a 1999 isolate of Flanders virus from Rhode Island. Likewise, no relationship could be demonstrated between RI-166, CT-114, Connecticut, or Flanders viruses by IFA test (data not shown). Based on these findings, we conclude that RI-166 is probably a new, unassigned vertebrate rhabdovirus. The name Rhode Island virus is proposed for this virus.

**Table 1:** Cross-reaction of CT-114 and RI-166 viruses with other selected rhabdoviruses by complement fixation test

Antigen	Hyperimmune ascitic fluid					
	Connecticut	New Minto	Sawgrass	Flanders	CT-114	RI-166
Connecticut	256/≥64^a^	0	128/32	0	0	0
New Minto	0	256/≥64	0	0	0	0
Sawgrass	16/32	16/32	1,024/64	0	0	0
RI 907-36	0	0	0	≥256/≥32	0	0
CT-114	0	0	0	0	256/64	0
RI-166^b^	0	0	0	0	0	128/≥8
RI-175 ^b^	0	0	0	0	0	128/≥8
^a^Reciprocal of ascitic fluid titer/reciprocal of antigen titer. ^b^RI-166 and RI 175 antigens were fluids from infected cell cultures.						

In CF tests, CT-114 HMAF reacted with five vesicular stomatitis serogroup antigens: Chandipura, Isfahan, Maraba, Jurona, and La Joya (Table 2). Antigenically, CT-114 was most closely related to Jurona and La Joya viruses. Both Jurona and La Joya viruses are tentative members of the genus *Vesiculovirus* (*14*–*16*). Based on the morphology and antigenic relationships of CT-114, we conclude that it is also a provisional member of the *Vesiculovirus* genus. The name Farmington is proposed for this new virus.

**Table 2:** Cross-reaction of CT-114 and RI-166 viruses and selected vesicular stomatitis serogroup viruses by complement fixation test

Antigen	Hyperimmune ascitic fluid						
	Chandipura	Isfahan	Maraba	Jurona	La Joya	CT-114	RI-166
Chandipura	256/≥32^a^	0	0	0	0	8/8	0
Isfahan	32/≥16	64/≥32	8/8	0	0	8/16	0
Maraba	8/≥8	0	512/≥32	0	0	8/16	0
Jurona	0	0	0	1,024/≥32	0	16/≥32	0
La Joya	0	0	0	0	512/≥32	16/≥32	0
CT-114	0	0	0	0	0	256/≥16	0
RI-166^b^	0	0	0	0	0	0	128/≥8

^a^Reciprocal of ascitic fluid titer/reciprocal of antigen titer. ^b^ RI-166 antigen was fluid from an infected Vero cell culture.

## Conclusion

The isolation of these new rhabdoviruses from birds demonstrates the value of direct culture for detecting new and unexpected viral agents. Rhode Island virus was initially isolated in Vero cells; Farmington virus was detected by intracerebral inoculation of newborn mice. To save time and reduce costs, many arbovirus diagnostic laboratories in the United States have stopped culturing field specimens and instead are using techniques such as antigen-capture enzyme-linked immunosorbent assay (*17*) or polymerase chain reaction (*18*–*20*) to detect viral antigens or nucleic acids in insect pools, blood, and tissue samples. While these newer techniques are rapid and quite sensitive, they detect only those viruses for which one has a capture antibody or a specific primer set. Furthermore, these techniques do not detect novel or unexpected viral agents nor antigenic or virulence changes in known viruses. A recent commentary (*21*) on the changing paradigm for arbovirus identification discussed these limitations of the more rapid molecular methods and stressed the importance of isolating viruses and obtaining phenotypic as well as genotypic information on them.

The isolation of Rhode Island virus from dead pigeons suggests that this virus may be an occasional avian pathogen. During the summer of 2000, a total of 335 birds, representing 31 avian species, were tested for virus at the CVBD. Rhode Island virus was isolated from 2 of 15 pigeons tested, suggesting that its host range may be restricted. Further experimental studies are needed to determine its pathogenesis and host range. In the northeastern United States, WNV, and to a lesser degree, EEEV, are the arboviruses usually associated with bird deaths (*3*,*22*). However, as surveillance for WNV continues and more dead birds are collected and cultured, other novel avian viral pathogens, such as Rhode Island virus, will probably be encountered.

At present, little is known about the ecology of Rhode Island or Farmington viruses. The ultrastructure and antigenic relationships of Farmington virus suggest that it is a novel vesiculovirus. The ability of Farmington virus to infect the *Cx. quinquefasciatus* cell line is also compatible with a vesiculovirus, since most of the rhabdoviruses in this genus are arthropod associated (*23*,*24*). Jurona and La Joya viruses, the vesiculoviruses most closely related antigenically to Farmington virus, were both isolated from New World mosquitoes. Jurona virus has been isolated from *Haemagogus* sp. and from a human in northern Brazil (*25*); La Joya was isolated from *Cx. dunni* in Panama (*13*).

Rhode Island virus is more intriguing. Its isolation from dead birds and its ability to infect mice (both newborn and adult) as well as Vero cells, are strong evidence that it is a vertebrate rhabdovirus. Yet its ultrastructure and relatively large size more closely resemble some of the plant rhabdoviruses (*26*). Further studies of this interesting new rhabdovirus and potential avian pathogen are warranted.
